# *N*-(Phenyl­sulfon­yl)acetamide

**DOI:** 10.1107/S1600536810015849

**Published:** 2010-05-08

**Authors:** B. Thimme Gowda, Sabine Foro, P. G. Nirmala, Hartmut Fuess

**Affiliations:** a Department of Chemistry, Mangalore University, Mangalagangotri 574 199, Mangalore, India; b Institute of Materials Science, Darmstadt University of Technology, Petersenstrasse 23, D-64287 Darmstadt, Germany

## Abstract

In the title compound, C_8_H_9_NO_3_S, the N—H bond is in an anti­periplanar conformation with respect to the C=O bond. The crystal packing is stabilized by N—H⋯O hydrogen bonds, generating *C*(4) chains propagating in [001].

## Related literature

Sulfonamide drugs contain the sulfanilamide moiety, see: Maren (1976 ▶). The propensity for hydrogen bonding in the solid state, due to the presence of various hydrogen bond donors and acceptors, can give rise to polymorphism, see: Yang & Guillory (1972 ▶). For the hydrogen-bonding preferences of sulfonamides, see: Adsmond & Grant (2001 ▶). For related structures, see: Gowda *et al.* (2008**a* ▶,b* ▶, 2009 ▶).
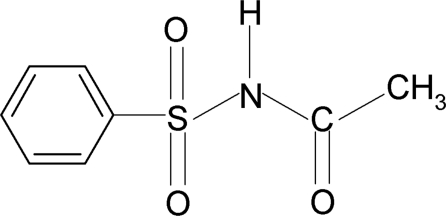


## Experimental

### 

#### Crystal data

C_8_H_9_NO_3_S
*M* *_r_* = 199.22
Tetragonal, 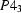

*a* = 7.9400 (5) Å
*c* = 15.288 (2) Å
*V* = 963.81 (15) Å^3^
*Z* = 4
Mo *K*α radiation
μ = 0.31 mm^−1^
*T* = 299 K
0.30 × 0.24 × 0.12 mm

#### Data collection

Oxford Diffraction Xcalibur diffractometer with a Sapphire CCD detector
Absorption correction: multi-scan (*CrysAlis RED*; Oxford Diffraction, 2009 ▶) *T*_min_ = 0.913, *T*_max_ = 0.964
2706 measured reflections
1401 independent reflections
1214 reflections with *I* > 2σ(*I*)
*R*_int_ = 0.014

#### Refinement

*R*[*F* ^2^ > 2σ(*F* ^2^)] = 0.051
*wR*(*F* ^2^) = 0.100
*S* = 1.30
1401 reflections
121 parameters
2 restraints
H atoms treated by a mixture of independent and constrained refinement
Δρ_max_ = 0.21 e Å^−3^
Δρ_min_ = −0.24 e Å^−3^
Absolute structure: Flack (1983 ▶), 378 Friedel pairs
Flack parameter: 0.11 (16)

### 

Data collection: *CrysAlis CCD* (Oxford Diffraction, 2009 ▶); cell refinement: *CrysAlis RED* (Oxford Diffraction, 2009 ▶); data reduction: *CrysAlis RED*; program(s) used to solve structure: *SHELXS97* (Sheldrick, 2008 ▶); program(s) used to refine structure: *SHELXL97* (Sheldrick, 2008 ▶); molecular graphics: *PLATON* (Spek, 2009 ▶); software used to prepare material for publication: *SHELXL97*.

## Supplementary Material

Crystal structure: contains datablocks I, global. DOI: 10.1107/S1600536810015849/bt5256sup1.cif

Structure factors: contains datablocks I. DOI: 10.1107/S1600536810015849/bt5256Isup2.hkl

Additional supplementary materials:  crystallographic information; 3D view; checkCIF report

## Figures and Tables

**Table 1:** Hydrogen-bond geometry (Å, °)

*D*—H⋯*A*	*D*—H	H⋯*A*	*D*⋯*A*	*D*—H⋯*A*
N1—H1*N*⋯O3^i^	0.85 (2)	2.02 (3)	2.823 (5)	156 (5)

Symmetry code: (i) 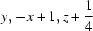.

**Fig. 1.**
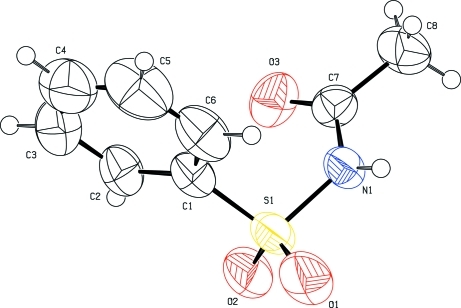
Molecular structure of the title compound, showing the atom labelling scheme. Displacement ellipsoids are drawn at the 50% probability level.

**Fig. 2.**
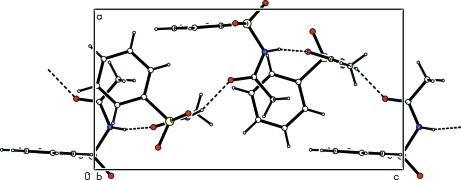
Molecular packing in the title compound. Hydrogen bonds are shown as dashed lines.

## supplementary crystallographic information

### Comment

Sulfonamide drugs contain the sulfanilamide moiety (Maren, 1976). The propensity for hydrogen bonding in the solid state, due to the presence of various hydrogen bond donors and acceptors can give rise to polymorphism (Yang & Guillory, 1972). The hydrogen bonding preferences of sulfonamides has also been investigated (Adsmond & Grant, 2001). The nature and position of substituents play a significant role on the crystal structures of *N*-(aryl)sulfonoamides (Gowda *et al.*, 2008*a,b*, 2009). As a part of studying the substituent effects on the structures of this class of compounds, the structure of *N*-(phenylsulfonyl)-acetamide (I) has been determined. The conformations of the N—H and C=O bonds of the SO_2_—NH—CO—C segment in the structure are anti to each other (Fig. 1), similar to that observed in *N*-(phenylsulfonyl)-2,2-dimethylacetamide (II)(Gowda *et al.*, 2009), *N*-(phenylsulfonyl)-2,2,2- trimethylacetamide (III)(Gowda *et al.*, 2008*b*) and *N*-(phenylsulfonyl)-2,2-dichloroacetamide (IV) (Gowda *et al.*, 2008*a*).

The C7—N1 bond in the C—SO_2_—NH—C segment of (I) is gauche [C7—N1—S1—O2 = 58.3 (5)°] with respect to the S1═O1 bond and anti with respect to the S1═O2 bond [C7—N1—S1—O1 = -172.6 (4)°]. The molecule in (I) is bent at the *S*-atom with a C1—S1—N1—C7 torsion angle of -58.8 (4)°, compared to the value of 67.1 (3)° in (II) and -66.3 (3)° in (IV),

The packing of molecules linked by N—H···O hydrogen bonds (Table 1) is shown in Fig. 2.

### Experimental

The title compound was prepared by refluxing benzenesulfonamide (0.10 mole) with an excess of acetyl chloride (0.20 mole) for about an hour on a water bath. The reaction mixture was cooled and poured into ice cold water. The resulting solid was separated, washed thoroughly with water and dissolved in warm dilute sodium hydrogen carbonate solution. The title compound was reprecipitated by acidifying the filtered solution with glacial acetic acid. It was filtered, dried and recrystallized from ethanol. The purity of the compound was checked by determining its melting point. It was characterized by recording its infrared spectra.

Rod like colorless single crystals were obtained from a slow evaporation of an ethanolic solution of the compound.

### Refinement

The H atom of the NH group was located in a difference map. Its coordinates were refined with a distance restraint of N—H = 0.86 (2) Å. The other H atoms were positioned with idealized geometry using a riding model with C—H = 0.93–0.96 Å. All H atoms were refined with isotropic displacement parameters  set to 1.2 times of the *U*_eq_ of the parent atom.

### Crystal data

**Table:**

C_8_H_9_NO_3_S	*D*_x_ = 1.373 Mg m^−^^3^
*M**_r_* = 199.22	Mo *K*α radiation, λ = 0.71073 Å
Tetragonal, *P*4_3_	Cell parameters from 1337 reflections
Hall symbol: P 4cw	θ = 2.6–27.9°
*a* = 7.9400 (5) Å	µ = 0.31 mm^−^^1^
*c* = 15.288 (2) Å	*T* = 299 K
*V* = 963.81 (15) Å^3^	Rod, colourless
*Z* = 4	0.30 × 0.24 × 0.12 mm
*F*(000) = 416	

### Data collection

**Table:**

Oxford Diffraction Xcalibur diffractometer with a Sapphire CCD detector	1401 independent reflections
Radiation source: fine-focus sealed tube	1214 reflections with *I* > 2σ(*I*)
graphite	*R*_int_ = 0.014
Rotation method data acquisition using ω and φ scans	θ_max_ = 26.4°, θ_min_ = 2.6°
Absorption correction: multi-scan (*CrysAlis RED*; Oxford Diffraction, 2009)	*h* = −5→9
*T*_min_ = 0.913, *T*_max_ = 0.964	*k* = −9→5
2706 measured reflections	*l* = −18→19

### Refinement

**Table:**

Refinement on *F*^2^	Secondary atom site location: difference Fourier map
Least-squares matrix: full	Hydrogen site location: inferred from neighbouring sites
*R*[*F*^2^ > 2σ(*F*^2^)] = 0.051	H atoms treated by a mixture of independent and constrained refinement
*wR*(*F*^2^) = 0.100	*w* = 1/[σ^2^(*F*_o_^2^) + (0.0165*P*)^2^ + 0.6175*P*] where *P* = (*F*_o_^2^ + 2*F*_c_^2^)/3
*S* = 1.30	(Δ/σ)_max_ = 0.022
1401 reflections	Δρ_max_ = 0.21 e Å^−^^3^
121 parameters	Δρ_min_ = −0.24 e Å^−^^3^
2 restraints	Absolute structure: Flack (1983), 378 Friedel pairs
Primary atom site location: structure-invariant direct methods	Flack parameter: 0.11 (16)

### Special details

**Table:**

Experimental. CrysAlis RED (Oxford Diffraction, 2009) Empirical absorption correction using spherical harmonics, implemented in SCALE3 ABSPACK scaling algorithm.
Geometry. All esds (except the esd in the dihedral angle between two l.s. planes) are estimated using the full covariance matrix. The cell esds are taken into account individually in the estimation of esds in distances, angles and torsion angles; correlations between esds in cell parameters are only used when they are defined by crystal symmetry. An approximate (isotropic) treatment of cell esds is used for estimating esds involving l.s. planes.
Refinement. Refinement of *F*^2^ against ALL reflections. The weighted *R*-factor wR and goodness of fit *S* are based on *F*^2^, conventional *R*-factors *R* are based on *F*, with *F* set to zero for negative *F*^2^. The threshold expression of *F*^2^ > σ(*F*^2^) is used only for calculating *R*-factors(gt) etc. and is not relevant to the choice of reflections for refinement. *R*-factors based on *F*^2^ are statistically about twice as large as those based on *F*, and *R*- factors based on ALL data will be even larger.

### Fractional atomic coordinates and isotropic or equivalent isotropic displacement parameters (Å^2^)

**Table:**

	*x*	*y*	*z*	*U*_iso_*/*U*_eq_	
C1	0.1027 (6)	0.4526 (6)	−0.0895 (3)	0.0558 (12)	
C2	0.1149 (6)	0.4033 (7)	−0.1756 (3)	0.0646 (14)	
H2	0.1204	0.2895	−0.1896	0.078*	
C3	0.1188 (7)	0.5227 (9)	−0.2413 (4)	0.0866 (18)	
H3	0.1238	0.4899	−0.2996	0.104*	
C4	0.1154 (8)	0.6897 (9)	−0.2194 (5)	0.099 (2)	
H4	0.1202	0.7704	−0.2635	0.119*	
C5	0.1051 (9)	0.7400 (8)	−0.1344 (7)	0.108 (3)	
H5	0.1017	0.8542	−0.1209	0.129*	
C6	0.0996 (7)	0.6210 (9)	−0.0676 (5)	0.0869 (19)	
H6	0.0939	0.6543	−0.0093	0.104*	
C7	0.4226 (6)	0.3105 (6)	0.0165 (3)	0.0550 (12)	
C8	0.5621 (6)	0.3530 (7)	0.0779 (4)	0.0792 (17)	
H8A	0.5555	0.2816	0.1285	0.095*	
H8B	0.5523	0.4686	0.0956	0.095*	
H8C	0.6684	0.3361	0.0492	0.095*	
N1	0.2655 (5)	0.3259 (5)	0.0517 (2)	0.0543 (10)	
H1N	0.250 (6)	0.373 (6)	0.1013 (19)	0.065*	
O1	−0.0383 (4)	0.3502 (6)	0.0542 (3)	0.0969 (14)	
O2	0.0878 (5)	0.1384 (5)	−0.0435 (2)	0.0849 (13)	
O3	0.4421 (5)	0.2652 (5)	−0.0584 (2)	0.0725 (11)	
S1	0.08988 (14)	0.30118 (19)	−0.00585 (8)	0.0633 (4)	

### Atomic displacement parameters (Å^2^)

**Table:**

	*U*^11^	*U*^22^	*U*^33^	*U*^12^	*U*^13^	*U*^23^
C1	0.048 (3)	0.059 (3)	0.060 (3)	0.004 (2)	−0.007 (2)	−0.015 (3)
C2	0.066 (3)	0.070 (3)	0.057 (3)	0.004 (3)	−0.008 (3)	−0.014 (3)
C3	0.089 (5)	0.098 (5)	0.073 (4)	0.002 (3)	−0.017 (3)	0.007 (4)
C4	0.088 (5)	0.088 (5)	0.122 (6)	−0.005 (4)	−0.026 (4)	0.023 (5)
C5	0.115 (6)	0.059 (4)	0.149 (7)	0.003 (4)	−0.028 (5)	−0.020 (5)
C6	0.084 (4)	0.084 (5)	0.093 (4)	−0.002 (3)	−0.017 (4)	−0.028 (4)
C7	0.046 (3)	0.057 (3)	0.062 (3)	0.004 (2)	0.005 (2)	0.004 (2)
C8	0.043 (3)	0.105 (4)	0.089 (4)	0.003 (3)	−0.005 (3)	−0.001 (4)
N1	0.041 (2)	0.079 (3)	0.043 (2)	−0.0046 (18)	0.0041 (18)	−0.017 (2)
O1	0.0412 (19)	0.173 (4)	0.077 (2)	−0.004 (2)	0.009 (2)	−0.017 (3)
O2	0.109 (3)	0.073 (3)	0.074 (2)	−0.029 (2)	−0.019 (2)	−0.012 (2)
O3	0.069 (2)	0.090 (3)	0.059 (2)	0.019 (2)	0.0191 (18)	0.0000 (19)
S1	0.0454 (6)	0.0888 (10)	0.0557 (6)	−0.0127 (6)	−0.0023 (7)	−0.0144 (7)

### Geometric parameters (Å, °)

**Table:**

C1—C6	1.378 (7)	C6—H6	0.9300
C1—C2	1.377 (6)	C7—O3	1.210 (5)
C1—S1	1.758 (5)	C7—N1	1.365 (6)
C2—C3	1.380 (7)	C7—C8	1.491 (6)
C2—H2	0.9300	C8—H8A	0.9600
C3—C4	1.367 (9)	C8—H8B	0.9600
C3—H3	0.9300	C8—H8C	0.9600
C4—C5	1.363 (9)	N1—S1	1.660 (4)
C4—H4	0.9300	N1—H1N	0.854 (19)
C5—C6	1.393 (10)	O1—S1	1.425 (4)
C5—H5	0.9300	O2—S1	1.415 (4)
			
C6—C1—C2	120.7 (5)	O3—C7—N1	121.1 (5)
C6—C1—S1	119.0 (5)	O3—C7—C8	124.7 (5)
C2—C1—S1	120.3 (4)	N1—C7—C8	114.2 (4)
C3—C2—C1	120.1 (5)	C7—C8—H8A	109.5
C3—C2—H2	120.0	C7—C8—H8B	109.5
C1—C2—H2	120.0	H8A—C8—H8B	109.5
C4—C3—C2	119.2 (6)	C7—C8—H8C	109.5
C4—C3—H3	120.4	H8A—C8—H8C	109.5
C2—C3—H3	120.4	H8B—C8—H8C	109.5
C5—C4—C3	121.2 (7)	C7—N1—S1	123.2 (3)
C5—C4—H4	119.4	C7—N1—H1N	122 (3)
C3—C4—H4	119.4	S1—N1—H1N	113 (3)
C4—C5—C6	120.2 (6)	O2—S1—O1	120.2 (2)
C4—C5—H5	119.9	O2—S1—N1	109.5 (2)
C6—C5—H5	119.9	O1—S1—N1	103.1 (2)
C1—C6—C5	118.6 (6)	O2—S1—C1	109.2 (2)
C1—C6—H6	120.7	O1—S1—C1	108.8 (3)
C5—C6—H6	120.7	N1—S1—C1	104.8 (2)
			
C6—C1—C2—C3	−2.0 (8)	C7—N1—S1—O2	58.3 (5)
S1—C1—C2—C3	177.7 (4)	C7—N1—S1—O1	−172.6 (4)
C1—C2—C3—C4	1.9 (8)	C7—N1—S1—C1	−58.8 (4)
C2—C3—C4—C5	−1.2 (11)	C6—C1—S1—O2	177.4 (4)
C3—C4—C5—C6	0.7 (12)	C2—C1—S1—O2	−2.3 (5)
C2—C1—C6—C5	1.5 (8)	C6—C1—S1—O1	44.4 (5)
S1—C1—C6—C5	−178.3 (5)	C2—C1—S1—O1	−135.3 (4)
C4—C5—C6—C1	−0.8 (10)	C6—C1—S1—N1	−65.3 (4)
O3—C7—N1—S1	−5.6 (7)	C2—C1—S1—N1	114.9 (4)
C8—C7—N1—S1	174.7 (4)		

### Hydrogen-bond geometry (Å, °)

**Table:**

*D*—H···*A*	*D*—H	H···*A*	*D*···*A*	*D*—H···*A*
N1—H1N···O3^i^	0.85 (2)	2.02 (3)	2.823 (5)	156 (5)

Symmetry codes: (i) *y*, −*x*+1, *z*+1/4.
